# Gut microbiota: a potential influencer of insomnia occurring after COVID-19 infection

**DOI:** 10.3389/fpsyt.2024.1423715

**Published:** 2024-07-23

**Authors:** Jiale Fang, Siwen Wang, Lijia Liu, Xiaoyi Zhang, Ruilong Liu, Xingchao Pang, Jiankun Cui, Jianshu Han, Xinyu Zhu

**Affiliations:** ^1^ The Third Affiliated Hospital, Heilongjiang University of Chinese Medicine, Harbin, China; ^2^ The Second Affiliated Hospital, Heilongjiang University of Chinese Medicine, Harbin, China; ^3^ The First Affiliated Hospital, Heilongjiang University of Chinese Medicine, Harbin, China

**Keywords:** COVID-19, insomnia, gut microbiota, probiotic bacteria, traditional Chinese medicine

## Abstract

The prevalence of insomnia has increased in recent years, significantly affecting the lives of many individuals. Coronavirus disease 2019 (COVID-19) infection has been found to have a substantial impact on the human gut microbiota (GM). Clinical studies have shown that the high prevalence, prolonged duration, and refractory treatment of insomnia symptoms following the COVID-19 pandemic may be related to the effect of COVID-19 infection on the GM. Therefore, the GM may be a potential target for the treatment of insomnia following COVID-19 infection. However, relevant studies have not been well-documented, and the GM has not been sufficiently analyzed in the context of insomnia treatment. Herein, we review the interaction between sleep and the GM, summarize the characteristics of COVID-19-induced abnormal changes in the GM and metabolites in patients with insomnia, and discuss potential mechanisms, including metabolic, immune, and neural pathways, by which these abnormal changes in the GM cause insomnia as well as the factors affecting the GM. Finally, we discuss the prospect of modulating the host GM community for the effective treatment of insomnia after COVID-19 infection and the need for further clinical studies.

## 1 Introduction

Coronavirus disease 2019 (COVID-19) has profoundly affected the lives and health of individuals worldwide. As the pandemic persists, severe acute respiratory syndrome coronavirus 2 (SARS-CoV-2) infections are observed to endure for extended periods (1). Insomnia emerges as a common clinical condition associated with post-acute COVID-19 syndrome (PACS). Previous studies have reported that the prevalence of sleep disorders in patients with COVID-19 infection ranges from 34% to 82% (2–5). A meta-analysis conducted in China revealed a 37% overall prevalence of insomnia symptoms in COVID-19 patients, with these symptoms persisting over time (4). Similarly, an online questionnaire administered during the COVID-19 pandemic in France indicated a prevalence of insomnia ranging from 3.9% to 22% (6). Another clinical study from Greece reported a 37.6% estimated prevalence of insomnia during the COVID-19 pandemic (7). Moreover, a large clinical meta-analysis summarized the prevalence of psychiatric symptoms in various populations from different countries, showing that insomnia occurred in 29.7% of the general public, 43.3% of pregnant women, and 58.4% of college students (8). Thus, worldwide reports indicate that insomnia resulting from COVID-19 infection is widespread.

Insomnia is a common clinical condition with multifactorial effects, intricately linked to cardiovascular, cerebrovascular, endocrine-metabolic, and neurological diseases, significantly impacting human health and quality of life (9–11). Several studies have demonstrated the association between gut microbiota (GM) and their metabolites with insomnia, capable of regulating sleep by influencing host brain function through signaling pathways mediated by the immune, neural, and endocrine systems (12, 13). A clinical study by Man et al. (12) revealed that patients with insomnia exhibited increased abundance of *Lactobacillus* and *Streptococcus*, along with decreased abundance of *Bifidobacterium*, *Gardnerella*, and *Streptococcus mutans* in the GM. Grosicki et al. (14) reported a positive correlation between sleep quality in young, healthy individuals and the diversity of the GM, as well as the Firmicutes/Bacteroidetes (F/B) ratio, but noted a negative correlation with the number of *Prevotella* and Bacteroidetes. Zhang et al. (15) observed a reduction in *Vibrio* spp. and *Vibrio butyric acidophilus* spp. in patients with depression and concurrent sleep disorders compared to those with depression alone. Furthermore, they noted significant variations in abundance of *Toxococcaceae*, *Dansoniaceae*, and *Dansoniaceae*.

Based on these findings, thorough examination is warranted to determine whether there are changes in the GM structure of patients with insomnia before and after COVID-19 infection. The prospect of leveraging these changes to develop new treatments for insomnia should be seriously considered. This may provide a basis for treating insomnia after COVID-19 infection by adjusting the GM structure and function. Previously, Alenazy et al. (16) reviewed the occurrence of anxiety, depression, panic disorder, and cognitive decline following COVID-19 infection in relation to changes in the GM. They proposed that probiotics may be useful as adjunctive therapy to enhance immunity and prevent PACS. Ghannoum et al. (17) analyzed the relationship between depression occurring after COVID-19 infection and the GM, proposing an approach based on modulating the GM to treat depression symptoms, including lifestyle modification and probiotic use. However, the variation in intestinal microflora and its primary mechanism of triggering insomnia after COVID-19 infection have not been summarized. Additionally, the use of traditional Chinese medical methods for regulating intestinal flora has not received considerable attention. Therefore, this study summarizes the changes in GM structural characteristics following the COVID-19 pandemic and explores the internal relationship between GM structural characteristics and insomnia treatment. Finally, potential methods that can effectively regulate GM structure and metabolism are recommended to improve the clinical treatment of insomnia following COVID-19 infection.

## 2 COVID-19 induces changes in the structure and metabolite profiles of intestinal microbial communities

**Figure 1:**
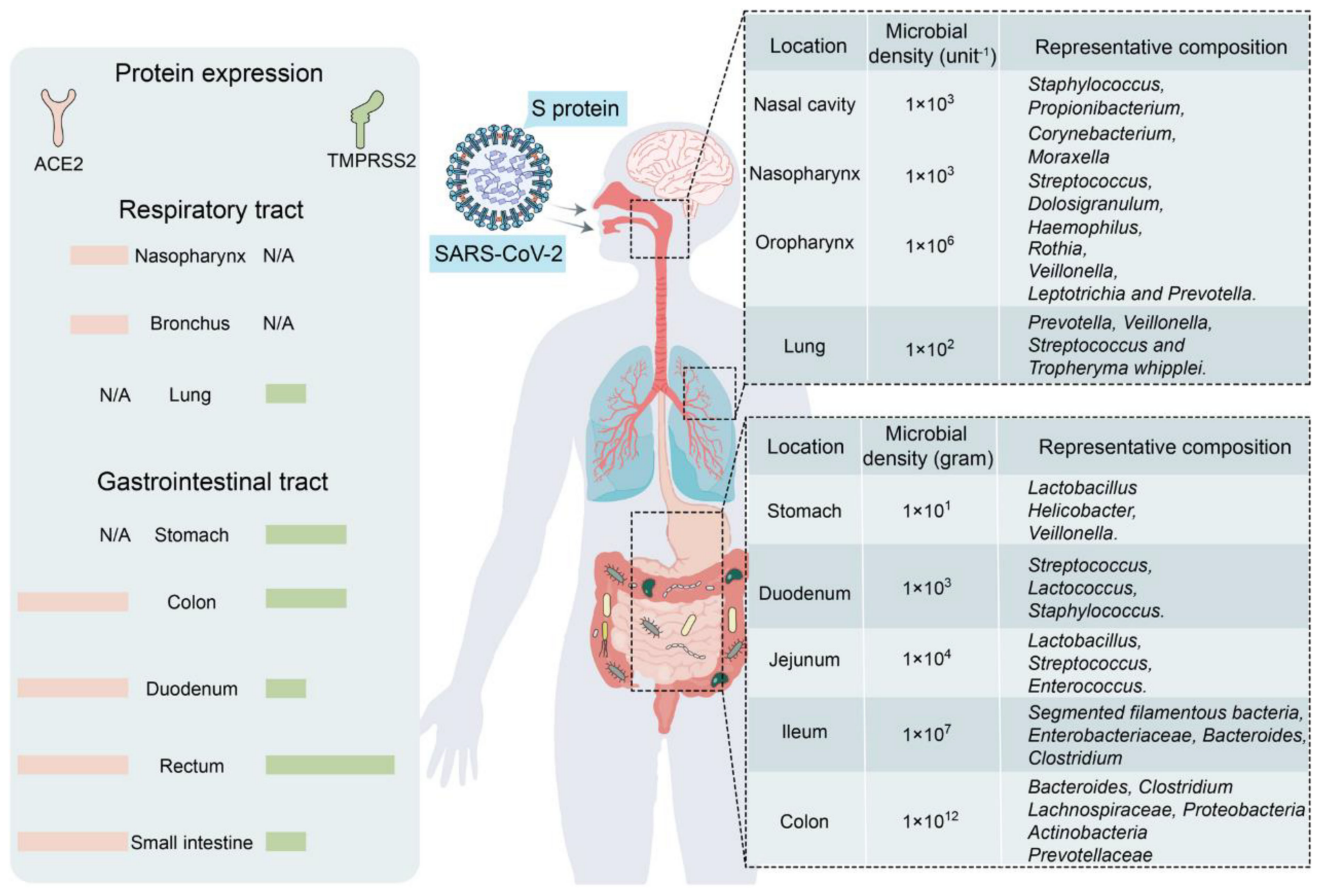
Primary habitats for human microbiota: the respiratory and gastrointestinal tracts as targets of SARS-CoV-2 infection (left: location of SARS-CoV-2 receptor ACE2 and TMPRSS2 expression; right: representative bacterial communities at different sites. Reproduced from **Figure 2**, 'Alterations in microbiota of patients with COVID-19: potential mechanisms and therapeutic interventions' by Wang et al., licensed under CC-BY (21).

The human GM is affected by COVID-19 infections with or without antibiotics, characterized by an enrichment of harmful pathogens and a reduction in beneficial microflora. These changes may be long-lasting and nonrecoverable (18, 19). Angiotensin-converting enzyme 2 (ACE-2), a functional host receptor for SARS-CoV-2 infection, is highly expressed in small intestinal cells (20), rendering the gastrointestinal system susceptible to SARS-CoV-2 infection (**Figure 1**). Some reports have shown that gastrointestinal symptoms occur in 11%–39% of patients with COVID-19 infection (22–25).

GM maintains gastrointestinal homeostasis and health in physiological states, and the high incidence of gastrointestinal symptoms observed in patients with COVID-19 infection associates the microbiota with SARS-CoV-2 infection. The likely mechanism may be that an inflammatory cytokine storm occurs during COVID-19 (26), releasing large numbers of interleukins (e.g., IL-6 and IL-1β), tumor necrosis factor-alpha (TNF-α), interferons (IFNs), and complement proteins (27), which leads to excessive immune cell activation that attacks the pathogen while causing damage to healthy cells, including intestinal microbes (28). Furthermore, elevated plasma levels of tight junction protein occludin, lipopolysaccharide binding protein, and *β*-glucan in patients with COVID-19 infection also indicate intestinal barrier dysfunction. The aforementioned factors would increase intestinal permeability, thereby affecting GM composition (29). Moreover, as a powerful immunomodulator in human health and disease, GM is involved in the host immune response to SARS-CoV-2 infection, such as promoting mucosal-associated T cell (MAIT) expression and increasing the secretion of deaminotyrosine (DAT), polysaccharide A (PSA), and vitamin A or retinoic acid, leading to a sustained GM impairment (21). In an *in vitro* study, Brogna et al. (30) reported that SARS-CoV-2 may alter the GM by directly infecting gut bacteria, similar to a phage. Furthermore, respiratory infections alter the composition and function of the intestinal microflora of the host through crosstalk between the GM and pulmonary system (also known as the “gut–lung axis”) (31, 32).

Some studies have shown that the bacterial diversity of the Lachnospiraceae, Ruminococcaceae, and Eubacteriaceae families is reduced in the intestines of patients with COVID-19 infection (33–35). In particular, the abundance of bacteria associated with short-chain fatty acid (SCFA) production decreases, and opportunistic pathogens from the Enterobacteriaceae family increase, as evidenced by a decrease in the abundance of *E. faecalis*, *E. funestus*, *E. faecium*, *E. ruminalis*, *L. pullorum*, and *E. roseus* and an increase in that of *E. enterococci*, *L. rosenbergii*, and *Lactobacillus* spp (33, 35–38). SARS-CoV-2 infection induces ACE-2 deficiency and downregulates B0AT1, a molecular ACE-2 chaperone, which reduces intestinal uptake of neutral amino acids (e.g., glutamine and tryptophan) required for the synthesis of 5-hydroxytryptophan (5-HT) (39). Moreover, the accumulation of neutral amino acids in the intestinal lumen results in changes in microflora, which can further induce sleep disorders by decreasing 5-HT secretion. Moreover, ACE-2 deficiency inhibits the activation of the intestinal mTOR pathway, decreasing AMP secretion, which subsequently affects GM composition (39). A study involving a few patients with COVID-19 infection who were not taking antibiotics showed a decrease in the genus *Alistipes* (belonging to the phylum Bacteroidetes), which is involved in the metabolism of tryptophan to 5-HT in the intestine (19).

Another study involving hospitalized patients in China showed that patients with COVID-19 infection had significantly lower intestinal bacterial diversity and abundance than healthy controls. Several SCFA-producing bacteria also showed a significant reduction, including *Agathobacter* spp., *Fusicatenibacter* spp., *Roseburia* spp., and *Ruminococcaceae UGC-013* (34). A clinical study involving patients with PACS showed that GM dysbiosis persisted for several months after COVID-19 virus clearance, and the relative abundance of *Bifidobacterium* and *Ruminal Streptococcus* members remained significantly reduced (*p* < 0.001) at the 6-month follow-up (40). These microbes are associated with SCFA production. Moreover, controls, such as reduced travel, home quarantine, wearing of masks, and increased sanitization during the COVID-19 pandemic, have been reported to affect the overall human GM (41). In conclusion, SARS-CoV-2 infection induces sleep disorders by disrupting the GM and their metabolites via multiple pathways, and continually summarizing and conducting new clinical studies to explore and confirm these relationships is necessary.

## 3 Intestinal flora and its influencing factors

### 3.1 Microorganisms

Approximately 90% of the cells in the human body consist of microorganisms, with the GM comprising the largest number and variety. The total cell number of GM in an adult is approximately 3.9 × 10^13^, encompassing approximately 10^4^ species (42), including bacteria, fungi, viruses, and single-celled eukaryotes. Bacteria, predominantly specialized anaerobes (comprising up to ≥99%), parthenogenetic anaerobes, and aerobes, dominate the intestinal community. Most of these bacteria belong to the phyla Firmicutes and Bacteroidetes, accounting for 90% of all members (43). The GM plays a crucial role in human metabolism, breaking down complex carbohydrates, proteins, and fats beyond the digestive tract, and metabolizing various small-molecule organic compounds such as SCFAs, alcohols, and ammonia (44). These metabolites not only provide energy for the body but also regulate the nervous and immune systems by influencing the physiological functions and gene expression of host cells (45, 46). The composition, diversity, and distributional traits of GM are not static after primary colonization but evolve dynamically with the host throughout the life cycle, adapting to different physiological and pathological states.

### 3.2 Common factors affecting GM

The human GM is primarily maintained by the release of specific factors (e.g., microRNAs) and nonspecific factors (e.g., antimicrobial peptides, mucus, and immunoglobulin A) by the host, contributing to its stability (47). Various factors including genes, diet, geography, disease, age, and even the mode of delivery can alter the GM (48–50). Additionally, self-metabolites such as SCFAs, bile acids, and choline metabolites can modulate GM composition (51, 52). Diet plays a crucial role in affecting GM diversity, influencing and maintaining GM homeostasis and circadian rhythms, and regulating circadian rhythms in the brain through the gut–brain axis (53). Human diet typically comprises three main nutrients: carbohydrates, proteins, and fats (54). The intake of different foods significantly impacts the structure and activity of trillions of microorganisms in the human gut (50, 55–58).

Vegetarian diets improve GM structure and are characterized by the predominance of flora that metabolize insoluble carbohydrates, such as *Ruminococcus*, *Roseburia*, and *Eubacterium* (59). Hou et al. (60) showed that older adults on a vegetarian diet had less severe COVID-19. David et al. (61) demonstrated that the GM can respond rapidly to altered diets, with an animal-based diet increasing the abundance of bile-tolerant microorganisms such as *Alistipes*, *Bilophila*, and *Bacteroides* while decreasing levels of Firmicutes bacteria that metabolize plant polysaccharides, including *Roseburia*, *Eubacterium rectale*, and *Ruminococcus bromii*. The possible mechanism is that animal diets contribute to the expression of bacterial genes encoding bile salt hydrolases, increasing intestinal deoxycholic acid (DCA) concentration, which induces microbial disturbances. In contrast, GM formed from plant-based diets inhibits the production of secondary bile acids (including DCA) and trimethylamine (TMA), helping to maintain a good intestinal barrier function and ensuring the stability and activity of the GM (62). Walker et al. (57) found that diets high in resistant starch (RS) increased the numbers of *R-ruminococci*, *Eubacterium*, and *Oscillospiracea*e in the gut. The abundance of Oscillospiraceae increased in RS and reduced carbohydrate weight loss (WL) dieters, whereas that of *Eubacterium* decreased in WL dieters. Cellulose and hemicellulose, two resistant carbohydrates present in plants, are associated with fiber-rich diets and the abundance of Bacteroidetes and Ruminococci in the human gut, with the final metabolite of Ruminococci being butyrate (63), positively correlated with sleep quality (64). Wang et al. (65) conducted a study administering a high-fiber diet to a patient with acute post-COVID-19 syndrome suffering from prolonged and severe gastrointestinal symptoms for 2 months. They found that the patient’s gastrointestinal symptoms improved, associated with an increase in SCFA-producing bacteria in the GM, primarily consisting of *Oscillibacter* sp., *Anaerofustis* spp., *Blautia* spp., and *Eubacterium hallii*.

Several small molecules also exert significant effects on the structure of the GM. Tea polyphenols (TPs) are common polyphenols in the diet and play a role in regulating sleep. Studies have shown that TPs can increase the abundance of intestinal flora, improve the composition of beneficial flora, and inhibit the growth of harmful bacteria (66). In one study, subjects were administered 0.4 g TP per dose three times a day for 4 weeks. The results showed a significant decrease in *Clostridium perfringens* and other *Clostridium* spp. in the intestinal tract during the period of tea polyphenol intake, while the abundance of *Bifidobacterium* spp. and the diversity of the intestinal microflora increased significantly (67). Melanoidins have a prebiotic-like effect on the human body, affecting the composition of the GM. In an experiment with mice fed melanoidin-enriched malt, a decrease in the relative abundance of *Dorea*, *Oscillibacter*, and *Alistipes* bacterial communities was observed, whereas that of *Lactobacillus*, *Parasutterella*, *Akkermansia*, *Bifidobacterium*, and *Barnesiella* increased, leading to higher production of SCFAs (68).

The rate of food digestion in the intestines also significantly impacts the structure of intestinal flora. When the amount of food ingested exceeds the digestion rate of the small intestine, escape or resistance to primary digestion occurs, thereby prolonging digestion time (69, 70). Roager et al. (69) demonstrated that a longer transport time of dietary material in the colon is associated with a higher abundance of intestinal microorganisms. The rate of digestion of dietary substances is influenced by several factors, such as diet type, rhythm, feeding cycle, exercise level, genetics, drugs (e.g., caffeine and alcohol), and psychological state (71, 72).

Because antibiotics continue to be widely used, their effects on the GM are gradually being recognized. A meta-analysis involving 2834 patients with COVID-19 infection showed a mean antibiotic utilization rate of 74.0% (73). Antibiotics eliminate pathogenic bacteria in the gut while destroying beneficial flora, thus affecting the ecological niches of microorganisms responsible for metabolic transformation in the gut wall (74). Antibiotic use results in a dramatic decrease in the α-diversity of adult intestinal flora, which does not fully recover within 6 months after completing treatment (75). Related findings indicate that the relative abundance of important members in the phyla Firmicutes, Bacteroidetes, and Actinobacteria has declined, most notably for *Faecalibacterium prausnitzii*, *Eubacterium* spp., *Roseburia* spp., *Anaerostipes* spp., and *Ruminococcus* spp (76). Reijnders et al. (77) showed that vancomycin reduced the abundance of the phylum Firmicutes, which is involved in SCFA and bile acid metabolism in the gut, while increasing the abundance of Enterobacteriaceae and *Enterococcus* sp. Patients treated with oral ciprofloxacin for urinary tract infections had decreased proportions of intestinal *Bifidobacterium*, *Alistipes*, *Faecalibacterium*, *Oscillospira*, *Ruminococcus*, and *Dialister*, along with an increased relative abundance of *Blautia*, *Eubacterium*, and *Roseburia*. Individuals treated with furaztoxin showed only a decrease in the genus *Clostridium* and an increase in the genus *Faecalibacterium* (78).

## 4 Interaction mechanisms between insomnia and intestinal microorganisms

### 4.1 Relationship between sleep and GM

The mechanism of human sleep comprises multiple physiological and neurological regulatory processes, including external clock regulation, internal rhythm regulation, cumulative sleep stress, and neuromodulation (79). The nervous system regulates the body’s sleep–wake cycle through a combination of neurotransmitters acting on different brain regions and neural circuits. Neurotransmitters are mostly produced by peripheral organs; thus, peripheral organs are important for the regulation of sleep-wakefulness. As the most important peripheral organ of the human body, the GM has communities and metabolite profiles that have a significant impact on sleep. A recent study suggested that the bidirectional action of the microbiota–gut–brain axis (MGBA) underlies the association between sleep and the GM (43). Wang et al. (80) reported that sleep duration is associated with GM diversity in preschool children and that the relative abundance of bifidobacteria in the intestinal tract was also high in children with high-quality sleep at night. Furthermore, another study demonstrated a positive correlation between GM diversity and total sleep time in adults (81). Besides, the metabolites produced by intestinal microorganisms, such as gamma-aminobutyric acid (GABA), dopamine, 5-HT, and melatonin, are capable of affecting sleep. Yu et al. (82) demonstrated that a decreased duration of sleep reduces the secretion of human defensin 5 in the host gut and disruption of GM structure, accompanied by a reduction in SCFA production. A study by Xie et al. (83) reported that individuals experiencing 2 days of partial sleep deprivation exhibited significant GM disruption. Increased food intake and insulin resistance, accumulation of adipose tissue with a systemic inflammatory response, and structural changes in the GM were observed in mice treated with chronic sleep fragmentation. These pathological changes were reproduced by transplanting the GM of these mice into normally sleeping germ-free mice (84). Alterations in the sleep–wake cycle resulted in irregular feeding times, which in turn caused changes in the GM composition. Abnormal feeding times were found to reduce the abundance of butyrate-producing bacteria in the mouse gut and colonic butyrate levels (85).

### 4.2 Action mechanisms of GM causing insomnia

**Figure 2:**
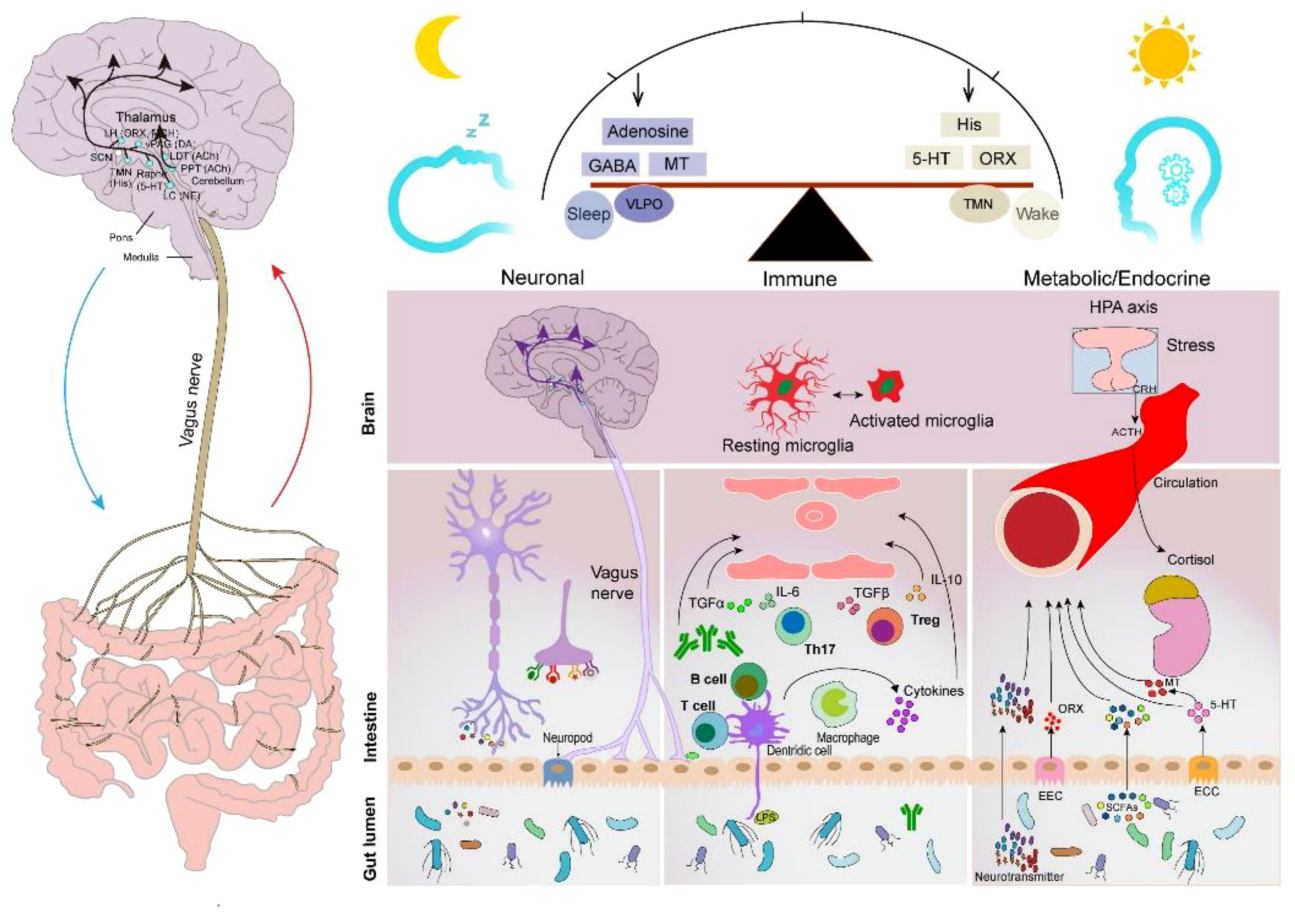
Sleep–GM interaction pathways (consisting mainly of neural, immune, metabolic, and endocrine pathways). Reproduced with permission from **Figure 3**, 'The microbiota-gut-brain axis in sleep disorders' by Wang et al., (88).

The human intestinal microflora, categorized from the phylum level, is primarily dominated by Bacteroidetes, Firmicutes, Ascomycetes, and Actinobacteria, which account for 99% of the total intestinal bacterial population (86), with approximately 3.3 million validated reference genes (87). It is the largest and most complex micro-ecosystem of the human body, capable of producing a series of metabolites with a wide range of bioactivities. Gut-generated signals communicate with sleep-related nuclei in the brain through the MGBA to regulate sleep and wakefulness (86). The main neurotransmitters produced by the GM that are associated with sleep signaling include melatonin, histamine, SCFAs, norepinephrine, GABA, and adenosine. They participate in sleep regulation through the following three pathways (**Figure 2**).

#### 4.2.1 Neuronal pathways

The brain and gut communicate with periventricular organs (mainly including the hippocampus, amygdala, and limbic cortex) in a bidirectional manner through the autonomic nervous system and ventricles (89). The vagus nerve (VN) is a major component of the parasympathetic nerves that are closely associated with sleep. Afferent nerves of the VN sense gut messages generated by the GM and its metabolites and transmit them to the central nervous system to generate appropriate responses that affect the human sleep–wake cycle (90). Furthermore, the efferent nerves of the VN contribute to the maintenance of GM community stability. The VN has been shown to promote inflammation reduction by regulating the levels of specialized proresolving mediators when subjected to peripheral stimuli, such as stress and inflammation (91), which is beneficial to intestinal epithelial health. For instance, one of the mechanisms could be that the VN communicates with the enteric nervous system to release nicotinic cholinergic signals to activate enteric glia cells (EGCs) (92), which can secrete S-nitrosoglutathione to increase the expression of tight junction proteins (93). Moreover, the VN can release acetylcholine (ACh) through the interaction of the enteric nervous system with the splanchnic nervous system. ACh inhibits the production and release of TNF and other proinflammatory cytokines using α7nAChR expressed on macrophages (94). Furthermore, a recent study showed that cholinergic signaling also promotes the biosynthesis of Alox15-dependent lipid mediators to accelerate the resolution of inflammation (95).

5-hydroxytryptamine (5-HT) is an inhibitory neurotransmitter synthesized and distributed in approximately 90% in enterochromaffin cells. Sleep disorders occur when 5-HT levels are reduced in the brain. 5-HT activates 5-HT3 receptors located in afferent fibers of the VN (96), and the central end of afferent fibers enters the brainstem through the solitary tract and synapses onto neurons of the nucleus tractus solitarius with glutamate as the primary neurotransmitter (97). Several studies have shown that some probiotic strains belonging to the Lactobacillus group, such as *L. rhamnosus* (98), *L. rohita* (99), *L. plantarum*, and *L. paracasei*, and *Bifidobacterium bifidum* (includes *B. longum* and *B. shortum*), are effective in increasing 5-HT levels in the hippocampus (100). Bercik et al. (101) found that after oral administration of *B. longum* NC3001, the behavior of mice with insomnia, induced by drinking dextran sodium sulfate, was normalized. This suggests that the anxiolytic effect involves vagal pathways in the gut–brain axis.

GABA is an inhibitory neurotransmitter that promotes body relaxation and reduces anxiety (102). Furthermore, it is the most widely studied GM metabolite. *Lactobacillus* and *Bifidobacterium* isolated from the human gastrointestinal tract can produce more than 20,000 μg ml^−1^ of GABA *in vitro* in the presence of sufficient and suitable substrates (103). Yu et al. (104) found that *Lactobacillus brevis* DL1–11 exhibited the highest GABA production capacity, and sleep duration was significantly prolonged in mice fed with fermented milk containing high doses of GABA. As a potential mechanism, following stimulation with GABA, the VN of the intestines transmits signals to the ventrolateral preoptic nucleus, nucleus raphe, blue plaque, and other structures in the brain, which in turn regulate the secretion of neurotransmitters and thus affect the sleep–wake cycle of the human body. Another study demonstrated that feeding *L. rhamnosus* to mice reduced their anxiety and insomnia/depression-like behavior, which may be mediated through central GABA receptor expression in the brain. These brain regions are associated with specific behaviors and exhibit concomitant changes in GABA Aα2 mRNA levels (105). Yamatsu et al. (106) administered 100 mg GABA capsules/day to patients with sleep disorders for 1 week and found a decrease in sleep latency and an increase in sleep duration involving non-rapid eye movement (NREM) (N1, N2, and N3/SWS).

#### 4.2.2 Immune pathways

The GM plays an important role in bidirectional communication between the brain, immune system, and gut (brain–gut–immune axis) (107). On the one hand, it stimulates innate immunity by activating lymphoid tissues located in the intestinal system; on the other hand, interactions between bacterial fragments and receptors (such as TLR9 and inflammasome) on the surface of epithelial and immune cells activate specific systemic and local immune responses (108). Sleep is a physiological state essentially related to the immune system (109). Sleep and immunity are bidirectionally linked, with immune system activation altering sleep, which in turn affects the immune system (110). The GM improves sleep by enhancing proinflammatory factors, such as IL-1 and TNF-α (111). Gut-derived immune mediators can be transmitted centrally through the circulatory system and vagal afferent pathways to affect sleep. In healthy men, overnight sleep deprivation increases TNF-alpha and C-reactive protein (112). TNF-α, an important cytokine of the immune system, promotes sleep by enhancing the 5-HT system (113). Lipopolysaccharides and SCFAs can influence the immune cell response and trigger microglia activation within the central nervous system (88). Microglia activation increases ceramide levels, resulting in an enhanced sleep drive, which, in turn, promotes sleep (114). Additionally, immunomodulatory factors IL-1β and IL-6 are closely related to sleep (115). Pulipati et al. (116) found that seven genera of the phylum Proteobacteria in the intestine, including *Sutterella*, *Oxalobacter*, *Desulfovibrio*, *Bilophila*, *Helicobacter*, *Pseudoalteromonas*, and *Succinivibrio*, were positively correlated with IL-6 levels.

#### 4.2.3 Metabolic and endocrine pathways

Neurotransmitters and metabolites produced by the GM and enteroendocrine cells, or enterochromaffin cells, regulate the sleep–wake cycle through the circulatory system. Melatonin is an important hormone that regulates the sleep–wake cycle, and patients with circadian rhythm disorders often exhibit decreased nocturnal plasma melatonin levels (117). One study showed a positive correlation between sleep quality and melatonin levels (118). Gut-derived melatonin is primarily converted from L-Tryptophan ingested by the gut through the serotonin pathway. Song et al. showed that the abundance of Roseburia in the intestine was positively correlated with melatonin expression in colonic mucosal tissue. This may be attributed to the metabolites of Roseburia, propionate, and butyrate, which promote the synthesis of intestinal melatonin by increasing 5-HT levels and upregulating arylalkylamine N-acetyltransferase expression via p-CREB (119).

Additionally, vitamin B6, also known as pyridoxine, consists of pyridoxine, pyridoxal, and pyridoxamine and is a key component of melatonin synthesis. Evaluation of the human GM genome for the B vitamin biosynthesis pathway revealed that 40%–65% of bacteria can synthesize this vitamin, making the GM an important B vitamin producer (120). Most species of the phyla Actinobacteria, Bacteroidetes, and Proteobacteria in the intestinal tract can synthesize pyridoxine (121). Gut-derived melatonin may affect sleep by attenuating melatonin receptor signaling in the brain through the gut-derived immune-mediated pathway; however, the exact mechanism requires further study.

SCFAs are produced by anaerobic bacteria or yeasts in the human gut through the fermentation of dietary fibers that the host cannot digest (122). Acetate, propionate, and butyrate are the most abundant SCFAs in the colon. Lactobacillaceae, Bifidobacteriaceae, Ruminalococcaceae, and *Clostridium* spp. are the primary producers of SCFAs in the gut (123). SCFAs improve the homeostasis and function of central neurons, contribute to serotonin biosynthesis by crossing the blood–brain barrier through the gut–brain axis (124), and link gut bacteria to brain sleep mechanisms (64). Furthermore, SCFAs have systemic and local anti-inflammatory and immunomodulatory functions in the gut. For instance, butyrate maintains epithelial barrier function by increasing TJs and AMPs, inducing regulatory T cells (Tregs), and controlling inflammation (125). Moreover, propionate contributes to the regulation of T cell production, HDACs activity, and TNF-α and IL-6 expression (126). However, most COVID-19-infected patients exhibit a deficiency in SCFA-producing flora. Magzal et al. (127) found that decreased SCFAs in the gut negatively impacted sleep duration and continuity in older adults. Acetate inhibits GABA secretion and increases glutamate–glutamine neurotransmitter levels via the hypothalamus (128). Studies have suggested that reduced GABA levels and increased glutamate–glutamine levels are associated with increased hyperarousal in patients with sleep disorders (129–131). Heath et al. (132) found that a higher proportion of propionate in fecal SCFAs is associated with a longer duration of uninterrupted sleep in infants. In a rodent study, oral administration of tributyrin (a butyrate prodrug) caused a nearly 50% increase in NREM in mice within 4 hours. Similarly, intraportal injection of butyrate caused a rapid and robust increase in the NREM of rats, with a 70% increase in 6 hours (64).

## 5 Treatment of insomnia after COVID-19 infection through GM regulation

**Figure 3:**
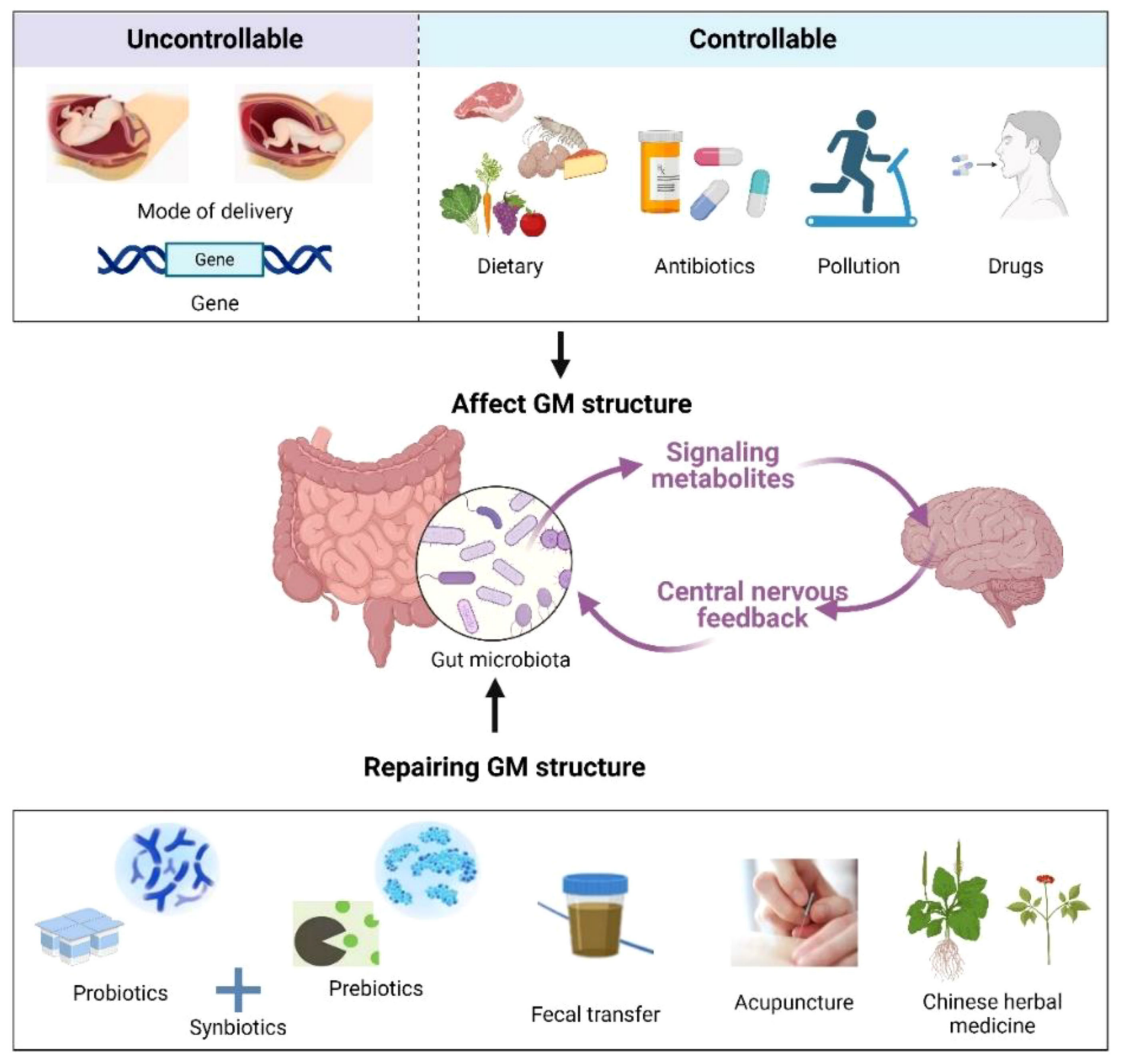
Factors affecting the GM (up) and remediation methods (down). Created with BioRender.com.

Targeting the GM has become an active area of study, and various therapeutics have entered the clinic for the treatment of diseases associated with COVID-19 infection. For example, supplementation with fermented vegetables was found to be associated with low mortality from COVID-19 infection, which may be due to enhanced antioxidant capacity induced by lactobacilli in the gut (133). A randomized controlled study by Gutiérrez et al. (134) revealed that probiotic supplementation relieved digestive symptoms and attenuated lung infiltrates in patients with COVID-19 infection, resulting in a reduction in patient nasopharyngeal SARS-COV-2 load. In recent years, as the relationship between GM, its metabolites, and sleep continues to be examined, new methods for insomnia treatment are being developed based on the regulation of GM, such as probiotics, prebiotics, fecal microbiota transplantation (FMT), and traditional Chinese medical methods [acupuncture and traditional Chinese medicine (TCM)] (**Figure 3**). These methods can circumvent the side effects often observed with Western sedatives. More importantly, they are beneficial to the overall health of the individual, such as improving immunity and metabolism.

### 5.1 Probiotics

Probiotics are widely used for treating various diseases and can influence the course of COVID-19 infections while maintaining GM health through various mechanisms, such as improving the antiviral response, producing antimicrobial peptides, and preventing secondary infections as well as exerting anti-inflammatory activity (135). Furthermore, probiotics exert immunomodulatory effects mainly by increasing IL-12; activating natural killer, Th1, and Th2 immune cells; and increasing IL-10 production to promote Treg cell production, thereby controlling inflammation (136, 137).

In a systematic review and meta-analysis of the effects of microbiota composition and probiotic supplementation on sleep quality by Santi et al. (138), probiotic supplementation significantly improved the sleep quality of patients compared with a placebo and reduced Pittsburgh Sleep Quality Index (PSQI) scores. Matsuda et al. (139) reported that oral administration of ergothioneine, a metabolite of *Lactobacillus reuteri*, to depressed rats prevented stress-induced sleep disorder and increased the duration of rapid eye movement (REM) sleep. Additionally, Miyazaki et al. (140) showed that administering *L. brevis* (a probiotic) prolonged both NREM and REM sleep in mice during the inactive phase (2 to 8 p.m.) compared with the control. Horvath et al. (141) demonstrated that a probiotic preparation containing nine strains of bacteria and three vitamin B compounds improved sleep quality and stress resistance in patients with insomnia caused by COVID-19 infection.

### 5.2 Prebiotics

Prebiotics, such as fructooligosaccharides (FOSs) and galactooligosaccharides (GOSs), are nutrients that are not easily digested but can cultivate beneficial intestinal microflora. For instance, *Lactobacillus* and *Bifidobacterium* can use prebiotics to produce SCFAs, GABA, and other sleep-friendly metabolites (142). FOSs and GOSs can assist immune factors, such as ILs, in both microbiota-dependent and independent manners, thereby exerting anti-inflammatory and immunomodulatory effects to maintain intestinal barrier integrity (143). For example, GOS-rich *Bifidobacteria* can induce IL-15 production by reversing goblet cell depletion (144). Moreover, prebiotics directly protect intestinal epithelial function, likely through the induction of upregulation of tight junction proteins (e.g., zonula occludens [ZO-1] and claudin-1) and downregulation of pro-inflammatory cytokines (e.g., IL-1β, IL-6, IFN-γ, and TNF-α) in intestinal epithelial cells (145). Burokas et al. (146) demonstrated that long-term probiotic FOS + GOS treatment exerted antidepressant and anxiolytic effects in mice and increased acetate and propionate concentrations in the cecum of mice. Thompson et al. (147) found increased levels of *L. rhamnosus* in the gut, which prolonged NREM sleep and reduced wakefulness in mice after administering a probiotic-enriched diet. They also demonstrated that such a diet prevented stress-induced reductions in microbial α-diversity. Schmidt et al. (148) showed that mice fed GOS had a significantly reduced salivary cortisol awakening response compared with mice fed FOS and a placebo (maltodextrin). The superior stress-reducing and sleep-promoting effects may result from GOS consumption in the gut, leading to increased production of SCFAs.

### 5.3 Synbiotics

Synbiotics are mixtures of probiotics and prebiotics, a combination aimed at enhancing the survival of probiotic microorganisms in the gastrointestinal tract. Synbiotics can be selectively utilized by the host GM and are considered potential GM modulators for immune functions (149). Supplementation with synbiotics has also been found to inhibit nuclear factor κ-B and reduce TNF-α production (150).

### 5.4 FMT

FMT involves implanting the GM flora from a healthy donor into a recipient, thereby restoring their normal GM community structure (151). The effect of FMT on psychiatric symptoms in patients with irritable bowel syndrome (IBS), functional diarrhea, and functional constipation has been examined, and the results indicate that FMT improves sleep quality and mood (152). Another study showed that treatment with a modified FMT approach was effective in relieving sleep disturbances, depression, anxiety, and gastrointestinal symptoms in IBS patients (153). Fang et al. (154) also demonstrated that FMT significantly improved sleep quality, alleviated anxiety and depression symptoms, and enhanced life quality in patients with chronic insomnia.

### 5.5 Acupuncture

Acupuncture is an important strategy for treating insomnia in TCM, serving as an effective complementary and alternative medicinal therapy. Several recent studies have suggested that acupuncture’s effects on insomnia treatment may be mediated through GM regulation. Gong et al. (155) conducted multiple acupuncture treatment sessions on COVID-19 patients experiencing insomnia, anxiety, nervousness, and irritability at the Wuhan Leishenshan Hospital. They found that these symptoms were alleviated following acupuncture. Hong et al. (156) used acupuncture to target Baihui, Sanyinjiao, and Shenmen points in p-chlorophenylalanine (PCPA)-induced insomnia mice, achieving efficacy comparable to that of zopiclone administration. They also observed an increase in the abundance of *Clostridium* XlVb, *Lachnospiracea incertae sedis*, *Anaerovorax*, *Oscillibacter*, *Pseudoflavonifractor*, and *Acetatifactor* in the intestinal tract of mice after acupuncture, thereby modulating the host immune response (e.g., triggering regulatory T cells and increasing IL-1β and IL-6 expression), which may be the primary mechanism underlying acupuncture’s role in improving sleep. In another study, Mongolian medical warm acupuncture (MMWA) was used to treat PCPA-induced insomnia in mice. The results showed that MMWA intervention significantly improved sleep quality, resulting in a decrease in the abundance of *Romboutsia* and an increase in the abundance of *Lactobacillus* and *Clostridium* XlVa in the mouse gut. Additionally, there was a decrease in acetylcholine and norepinephrine levels and an increase in 5-HT and GABA levels in mouse serum (157).

### 5.6 TCM

TCM is a medicine made from plants, animals or minerals based on the traditional Chinese medical theory, and it has been clinically used to treat insomnia for over 2000 years and also influences the composition and metabolism of GM (61). TCM contains alkaloids, flavonoids, glycosides, organic acids, and phenylpropanoids, which contribute to GM regulation (158). Specifically, it promotes the growth of beneficial bacteria and the production of SCFAs, inhibits the overgrowth of harmful bacteria, and maintains the immune barrier function of the intestinal mucosa (159). Wang et al. (160) demonstrated that Bailemian (a commercial TCM) alleviates PCPA-induced insomnia symptoms in mice. After 10 days of Bailemian treatment, GABA levels in the brain and colon of mice were elevated. There was an increased abundance of the phylum Verrucomicrobia in the gut, whereas the phylum Firmicutes decreased, indicating that Bailemian plays a role in balancing the GM structure. Perimenopausal insomniac women were administered Tianwang Buxin Granules (a commercial TCM) for four consecutive weeks to regulate intestinal flora disorders. This treatment increased the abundance of Bacteroides, fecal bacteriophages, and *F. prausnitzii*, while decreasing the abundance of *Roseburia faecis*, *Ruminococcus*, *Prevotella copri*, *Fusicatenibacter saccharivorans*, and *Blautia obeum* (161). In another study, after taking Suanzaoren Decoction for 2 weeks, insomnia patients exhibited a significant decrease in Chinese Medicine Symptom Score and PSQI, and an increase in the number of *Lactobacillus* and *Bifidobacterium* in the gut (162). Zhao et al. (163) found that the Buzhong Yiqi decoction significantly reduced the abundance of inflammatory pathogenic bacteria (i.e., Epsilonbacteraeota and Spirochaetes) in the gut of patients with insomnia, suggesting that this TCM improves insomnia by reducing the intestinal immune-inflammatory response.

## 6 Conclusions

SARS-CoV-2 infection can cause widespread and long-lasting changes in the host GM, mainly characterized by a decrease in beneficial flora and an enrichment of harmful pathogens. This review summarized numerous observational studies to date, which have preliminarily demonstrated that GM disorder is a major causative factor for insomnia after COVID-19 infection. It explored specific mechanisms, influencing factors, and currently feasible prevention and treatment options, providing a basis for treating insomnia after COVID-19 infection by modifying GM structure and function. It is recommended that patients with insomnia after SARS-CoV-2 infection regulate their dietary structure and lifestyle, reduce the unnecessary application of antibiotics, and promote appropriate intake of probiotics, prebiotics, and synbiotics to prevent or alleviate insomnia symptoms caused by COVID-19. If necessary, the FMT method can also be considered. Furthermore, the application of traditional Chinese medical methods to regulate the GM shows promise as a new approach for preventing and treating COVID-19-induced insomnia; however, further clinical studies are required to improve it. Although some progress has been made in the study of the interrelationship between sleep and the GM, many undiscovered mysteries about the GM await exploration. With the development of macro-genomics, metabolomics, viral immunology, and novel microbiome therapies, the clinical potential of using GM to treat insomnia will continue to expand.

## Author contributions

JF: Writing – original draft, Methodology, Investigation, Conceptualization. SW: Writing – original draft, Investigation, Data curation. LL: Writing – original draft, Visualization, Investigation. XiaZ: Writing – review & editing, Visualization, Investigation. RL: Writing – review & editing, Investigation. XP: Writing – review & editing, Investigation. JC: Writing – review & editing, Conceptualization. JH: Writing – review & editing, Investigation. XinZ: Writing – review & editing, Supervision.
